# Novel Design of Eco-Friendly High-Performance Thermoplastic Elastomer Based on Polyurethane and Ground Tire Rubber toward Upcycling of Waste Tires

**DOI:** 10.3390/polym16172448

**Published:** 2024-08-29

**Authors:** Maoyong He, Ruiping Li, Mingzheng Hao, Ying Tao, Peng Wang, Xiangcheng Bian, Haichun Dang, Yulong Wang, Zhenzhong Li, Tao Zhang

**Affiliations:** 1 Department of Materials Engineering, Taiyuan Institute of Technology, Taiyuan 030008, China; 2120700836@tit.edu.cn (P.W.); bianxiangcheng@tit.edu.cn (X.B.); danghc@tit.edu.cn (H.D.); wangyl@tit.edu.cn (Y.W.); lizhenzhong@tit.edu.cn (Z.L.); zhangt@tit.edu.cn (T.Z.); 2 College of Materials Science and Engineering, Northeast Forestry University, Harbin 150040, China; liruiping1009@nefu.edu.cn; 3 College of Material, Chemistry and Chemical Engineering, Hangzhou Normal University, Hangzhou 311121, China; 2023112009072@stu.hznu.edu.cn

**Keywords:** ground tire rubber, polyurethane elastomer, heat build-up, wet-skid resistance

## Abstract

Waste rubber tires are an area of global concern in relation to reducing the consumption of petrochemical products and environmental pollution. Herein, eco-friendly high-performance thermoplastic polyurethane (PU) elastomers were successfully in-situ synthesized through the incorporation of ground tire rubber (GTR). The excellent wet-skid resistance of PU/GTR elastomer was achieved by using mixed polycaprolactone polyols with Mn = 1000 g/mol (PCL-1K) and PCL-2K as soft segments. More importantly, an efficient solution to balance the contradiction between dynamic heat build-up and wet-skid resistance in PU/GTR elastomers was that low heat build-up was realized through the limited friction between PU molecular chains, which was achieved with the help of the network structure formed from GTR particles uniformly distributed in the PU matrix. Impressively, the tanδ at 60 °C and the DIN abrasion volume (Δrel) of the optimal PU/GTR elastomer with 59.5% of PCL-1K and 5.0% of GTR were 0.03 and 38.5 mm^3^, respectively, which are significantly lower than the 0.12 and 158.32 mm^3^ for pure PU elastomer, indicating that the PU/GTR elastomer possesses extremely low rolling resistance and excellent wear resistance. Meanwhile, the tanδ at 0 °C of the above-mentioned PU/GTR elastomer was 0.92, which is higher than the 0.80 of pure PU elastomer, evidencing the high wet-skid resistance. To some extent, the as-prepared PU/GTR elastomer has effectively solved the “magic triangle” problem in the tire industry. Moreover, this novel research will be expected to make contributions in the upcycling of waste tires.

## 1. Introduction

With the development seen in the automotive industry, global tire production is constantly increasing, and it is expected to reach 26.65 billion units in 2027 [1]. It is widely known that most rubber tires are typical mixtures composed of numerous compounds, such as rubber (including natural rubber and synthetic rubber), polymer cord fabric, carbon black, etc., most of which are petrochemical products [2]. Obviously, the manufacturing and use of traditional rubber tires often necessitate massive energy consumption [3]. Moreover, some traditional disposal routes of waste tires, including energy recovery, landfill, etc., can easily cause serious environmental pollution [4,5]. In order to achieve sustainable development, numerous efforts focusing on the “green tire”, such as rubber replacement [6], silica reinforcement [7,8,9], reversible cross-linking [10,11,12], polyurethane tires [13] and so on, have been carried out to solve the obstacles above.

In contrast to traditional rubber tires, thermoplastic polyurethane tires possess excellent wear resistance, mechanical strength, corrosion resistance, etc., inciting widespread interest in automobile tires. Due to the absence of additives such as carbon black, zinc oxide, vulcanizing agents, etc., the preparation of polyurethane tires effectively avoids the intensive consumption of petrochemical products and limits the pollution caused by rubber tire debris [14,15]. However, serious heat build-up is generated in polyurethane elastomers under high-speed dynamic loads, which massively delays the industrialization of high-performance polyurethane automobile tires.

It is found that the degree of microphase separation in polyurethane elastomers is closely related to its dynamic heat generation. Therefore, researchers have conducted a lot of research work from the points of views of polyurethane formulation design and additive modification, attempting to solve the above problems. On the one hand, some research works focus on using isocyanates [16,17] and chain-extenders [18,19], these being of high rigidity with highly symmetrical molecular structures, to synthesize polyurethane elastomers with a high degree of microphase separation, thus weakening the heat build-up of polyurethane through reducing the internal friction of molecular chains. On the other hand, a chemically micro-crosslinked structure is also incorporated into the polyurethane using functional micro/nano-particles [20,21] and triol, e.g., trifunctional poly-propylene glycerol (like trifunctional poly-propylene glycerol and trimethylolpropane) [22,23,24,25], endowing the final products with lower heat build-up. The micro-crosslinked structure in polyurethane could efficiently restrict the movement of macromolecular chains, thereby greatly reducing the heat build-up generated by friction among the chains under dynamic loads.

These chemically crosslinked points act as rigid points to some extent; however, they cannot effectively relax the local stress at the interface caused by the rotation and sliding of molecular chains. Conversely, the great local stress concentration will exacerbate the sliding motion of the molecular chains, further generating much frictional heat under high-speed alternating loads. Moreover, the frictional interaction between the rigid cross-linking points and polyurethane molecular chains also causes dynamic hysteresis loss after blending with high-mass nanofillers [26,27,28,29,30]. Although these use of excessive chemical crosslinking agents can significantly limit the movement of molecular chains and reduce dynamic heat generation, the resulting polyurethane elastomers will lose their thermos-plasticity and dissolvability, which goes against the sustainable development strategy.

Based on the macromolecular assembly strategy, Qin X et al. [31] innovatively prepared high-performance polyurethane elastomers with a high degree of microphase separation using hydroxy-terminated polybutadiene as polyols, exhibiting extremely low dynamic heat generation, in which a crosslinked structure was introduced into soft segments via controlled radiation. Coincidentally, polyurethane elastomers with low dynamic heat build-up were successfully prepared in our previous research based on the crosslinking strategy [32], wherein ground tire rubber (GTR) particles were employed as the “elastic crosslinked points”; however, the incorporation of GTR reduces the degree of microphase separation of polyurethane. Unfortunately, the wet-skid resistance of the above-mentioned thermoplastic elastomers cannot meet the requirements of polyurethane tires.

Fortunately, a high wet-skid resistance in polyurethane elastomer was achieved by the reduction in the average molecular weight of soft segments through the mixing of polycaprolactone (PCL) polyols, and the corresponding high dynamic heat build-up was alleviated via the network structure formed by the GTR particles that were evenly distributed in the PU matrix in this paper. The aim here is to further investigate the effects of the relative molecular weight of polyols on the microphase structure of PU/GTR elastomers with 5.0% GTR, in order to synthesize high-performance PU/GTR elastomers simultaneously possessing high wet-skid resistance, low heat build-up, and excellent mechanical properties. The interfacial strength and degree of microphase separation of PU/GTR elastomers were investigated through X-ray photoelectron spectroscopy (XPS) analysis and FTIR spectroscopy (FTIR) analysis. Further, the effect of PCL-1K content on the aggregation structure (such as microstructure of hard and soft segments, distribution of GTR particles and degree of microphase separation) of PU/GTR elastomers was discussed using a small-angle X-ray scattering (SAXS) test, differential scanning calorimetry (DSC) characterization and rheological measurements. Combined with the results of stress relaxation tests, the mechanisms endowing high wet-skid resistance and low heat build-up were ultimately revealed. Notably, this work is expected to provide a practical strategy for the high-value utilization of waste tires and the sustainable development of polyurethane green tires.

## 2. Materials and Methods

### 2.1. Materials

PCL polyols containing two kinds of PCL-1K (Mn = 1000 g/mol) and PCL-2K (Mn = 2000 g/mol) were purchased from the Daicel Corporation. 4,4′-diphenyl-methane diisocyanate (MDI) and 1,4-butanediol (BDO) were supplied by Huatian Rubber & Plastic technology Co., Ltd. (Yantai, China). The GTR with the particle size distribution shown in Figure S1, produced by grinding off-the-road tires, was supplied by Hongrui Rubber Co., Ltd. (Dujiangyan, China).

### 2.2. The Synthesis of the PU/GTR Thermoplastic Elastomers

**Scheme 1:**
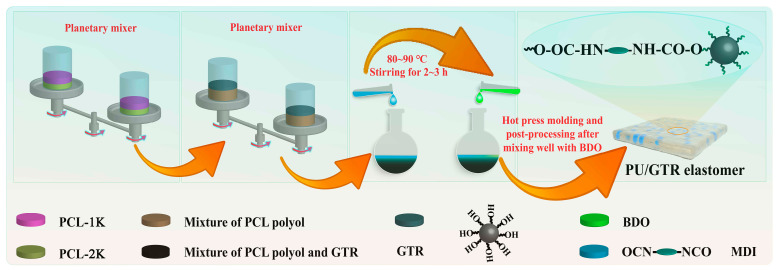
The synthesis process of PU/GTR thermoplastic elastomers.

Firstly, the PCL polyols were evenly mixed using a planetary vacuum mixer (MV-1000, Marath Technology Co., Ltd. (Shenzhen, China)) at 1000 rpm. Then the GTR and PCL polyols were also uniformly mixed using the planetary vacuum mixer before adding MDI to synthesized prepolymer (as shown in Scheme 1). It should be noted that the reaction time for prepolymer synthesis was also continuously extended, from 2.5 h to 4 h, with the increase in PCL-1K content. Immediately, the prepolymer was prepared by stirring the mixture of polyols and GTR, as well as MDI, in a three-necked flask. Finally, the PU/GTR thermoplastic elastomers were obtained via hot-pressing under 100 °C for 0.5 h and post-treatment at the same temperature for 24 h after mixing the prepolymer and BDO with the planetary mixer under a vacuum environment. The formulations used to synthesize the PU/GTR elastomer were made at a 1:1 molar ratio of NCO groups and OH groups, whereby the OH groups were from the GTR surface, BDO and PCL polyol (as shown in Table 1). Specifically, the content of OH group on the GTR surface was 5.5 mmol/g. The method of preparation of the pure polyurethane elastomer (PUE) without adding GTR was the same as in the above method.

### 2.3. Characterization

#### 2.3.1. Dynamic Mechanical Analysis (DMA)

The dynamic analyzer (Q800, TA Instruments, New Castle, PA, USA) was used to measure the dynamic mechanical property of PU/GTR thermoplastic elastomers, with the strain and the frequency being 1% and 10 Hz, respectively. The testing temperature range was from −80 °C to 150 °C at a heating rate of 3 K/min. In addition, the dimensions of tension-molded samples were 20 × 4 × 2 mm^3^.

#### 2.3.2. Mechanical Test

The mechanical properties were assessed on the universal testing machine (AI-7000M, GOTECH Testing Machines (Dongguan) Co., Ltd., Dongguan, China) with a crosshead speed of 500 mm/min according to ISO 37:2005 [33]. The testing samples with a thickness of 2 mm were dumbbell-shaped specimens (Type 2). The Shore A durometer was used to measure the hardness of the PU/GTR thermoplastic elastomers according to ISO 7619-1:2010 [34].

#### 2.3.3. Abrasion Loss Test

The abrasion loss of the PU/GTR elastomers was characterized through the DIN abrader (GT-7012-D, GOTECH Testing Machines (Dongguan) Co., Ltd., Dongguan, China) according to ISO 4649:2002 [35]. The testing load and sliding speed were 10 N and 0.32 m/s, respectively. Additionally, the DIN abrasion volume (Δrel) was averaged over at least five specimens.

#### 2.3.4. XPS Analysis

The XPS analysis was performed on a spectrometer (ESCALAB 250Xi, ThermoFisher Scientific, Waltham, MA, USA) using a focused monochromatic Al Kα. The operation condition was preset at 12.5 kV and 16 mA. The C1s peaks were fitted using the 20% Lorentzian–Gaussian function.

#### 2.3.5. FTIR Analysis

The spectra of PU/GTR thermoplastic elastomers were recorded on an infrared spectrometer (Nicolet iS50, ThermoFisher Scientific, Waltham, MA, USA) from 4000 cm^−1^ to 600 cm^−1^ at a resolution of 4 cm^−1^ over 64 scans. Note that PU/GTR elastomers were tested using attenuated total reflectance mode, while GTR samples (preparation provided in Supplementary Materials) were tested using ordinary infrared mode.

#### 2.3.6. SAXS Analysis

The microphase structure was characterized by the SAXS system (SAXSpoint 2.0, Anton Paar, Graz, Austria) at room temperature (25 °C). The X-ray wavelength was 0.154 nm, emitted by a Cu Kα1 X-ray source. A Pilatus 300K was used as the detector with a sample-to-detector distance of 541 mm, which was calibrated by Silver Behenate. The two-dimensional (2D) SAXS pattern was collected during heating with an acquisition time of 5 s. The sample was continuously heated from 25 to 180 °C, and the heating rate was 5 K/min.

#### 2.3.7. DSC Analysis

Differential scanning calorimetry (DSC) analysis was conducted on a thermal analyzer (Q20, TA Instruments, New Castle, PA, USA) under nitrogen atmosphere. The specimens (about 10 mg) were cooled down to −80 °C at a rate of 10 K/min, equilibrated for 10 min, and then heated to 230 °C at the rate of 10 K/min.

#### 2.3.8. Rheological Measurements

The rheological properties were measured using a rotary rheometer (ARES-G2, TA Instruments, New Castle, PA, USA) with a parallel plate of 25 mm in diameter. The frequency sweep range was from 0.01 to 100 Hz under a strain of 1.0%. Meanwhile, the test temperatures were 180, 190 and 200 °C, respectively.

#### 2.3.9. Stress Relaxation Test

The stress relaxation characterizations of PU/GTR elastomers were also conducted on the dynamic analyzer (Q800, TA Instruments, New Castle, PA, USA). Rectangular specimens (20 × 4 × 2 mm^3^) were heated to 80 °C at a heating rate of 3 K/min, equilibrated for 5 min, and then stretched to a strain of 7%. Then, the values of stress were recorded during the time which stress relaxation occurred under the same strain.

## 3. Results and Discussion

### 3.1. Mechanical Properties and Wear Resistance of PU/GTR Thermoplastic Elastomers

**Figure 1:**
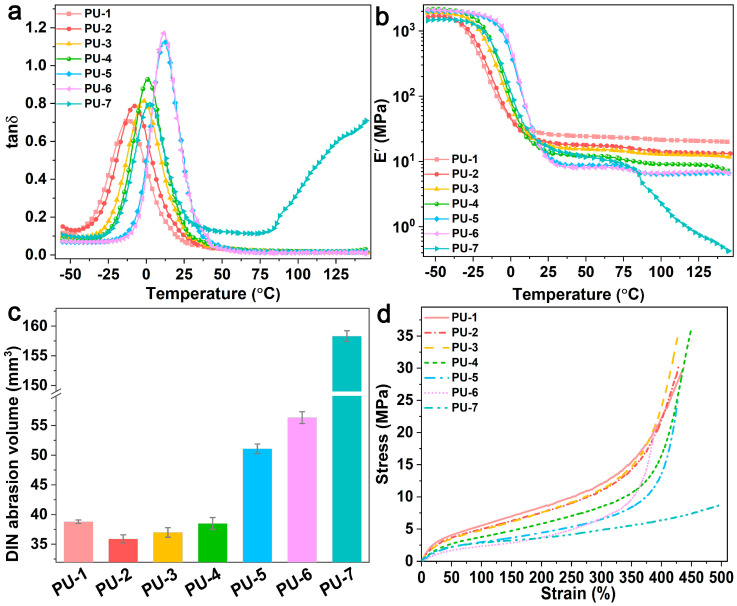
tanδ versus temperature curves (**a**), E′ versus temperature curves (**b**), DIN abrasion volume (**c**) and representative stress–strain curves (**d**) for PU/GTR thermoplastic elastomers.

The dynamic mechanical properties of PU/GTR thermoplastic elastomers are shown in Figure 1a,b. When the PCL-1K content increased, the distance between hard domains and the regularity of hard segments decreased, which significantly reduced the thermal activity of soft segments. Thus, the T_g_ values of soft segments were enhanced with increasing PCL-1K contents, increased from −11.7 to 12.0 °C (Table 2), which is consistent with the phenomenon seen in the literature [36]. Obviously, the increase in T_g_ is conducive to enhancing the tanδ at 0 °C of PU/GTR thermoplastic elastomers with a T_g_ much lower than 0 °C (Figure 1a). Furthermore, the possible network structure, which refers to the uniform distribution of GTR particles in the PU matrix in terms of network, also has a strong constraining effect on the soft segments, which makes the thermal transformation of the soft segments more difficult, resulting in a higher tanδ of the PU/GTR elastomer than of the pure PUE (PU-7). Consequently, the tanδ at 0 °C of the PU/GTR elastomer with 59.5% PCL-1K (PU-4) was as high as 0.92. Generally, the tanδ at 0 °C is always used to characterize the wet-skid resistance of tires [31]. The larger the tanδ at 0 °C is, the slower the dissipation rate of the accumulated heat generated by the tire driving on a wet road will become, which objectively causes strong friction between the tires and the ground, making the driving safer [37]. Compared with the polymeric materials used in the literature [13,31,37], of which the tan δ at 0 °C is generally less than 0.8, the PU-4 elastomer possesses an excellent wet-skid resistance. When the PCL-1K content is higher than 80%, the distance between hard domains and the orderliness of hard domains further decrease, resulting in a reduction in the degree of microphase separation in PU/GTR elastomers. As a consequence, the resulting stronger interaction between hard and soft segments significantly restricts the movement of soft segments, thus causing the PU/GTR elastomers to exhibit a much higher T_g_ (higher than 0 °C), which in turn reduces the tanδ at 0 °C (as shown in Table 2).

It is also obvious that the values of tanδ at 60 °C of PU/GTR elastomers are lower than those of pure PUE, and they remained at about 0.03 as the testing temperature was increased from 60 to 145 °C (as shown in Figure 1a and Table 2), evidencing that the PU/GTR elastomers display extremely low dynamic heat build-up. In general, the activity of molecular chains in soft segments will be reinforced as the temperature gradually increases, leading to an increase in heat build-up caused by the frication of molecular chains. Thus, the low dynamic heat generation of PU/GTR elastomers could be attributed to the network structure via the GTR particles in the PU matrix. The network structure significantly weakens the movement of polyurethane molecular chains via chemical bonds, van der Waals forces, and hydrogen bonds, especially for the chains in soft segments, thereby endowing the PU/GTR elastomers with lower dynamic heat build-up.

When the testing temperature is increased from 35 to 130 °C, the storage dynamic modulus (E′) of PU/GTR elastomers also remains almost constant, and it presents a gradual decreasing trend as the PCl-1K content increases, as displayed in Figure 1b. The E′ of pure PUE shows a sharp decreasing trend when the temperature is higher than 80 °C, which may be due to the serious mechanical hysteresis, causing an accelerated melting transformation behavior of the hard domains with lower regularity (discussed in detail in the following text). Hence, it is the strong reinforcement effect of the network structure that endows the final PU/GTR elastomers with a stable dynamic mechanical modulus. In a word, PU/GTR elastomers not only show low dynamic heat build-up, but also have a high and stable dynamic storage modulus.

The abrasion behavior of PU/GTR thermoplastic elastomers is determined in terms of DIN abrasion volume (Δrel), with the results shown in Figure 1c. Obviously, the addition of GTR improves the abrasion resistance of PU/GTR elastomers. Notably, the Δrel of the PU/GTR elastomer (PU-4) is 38.5 mm^3^, possibly attributable to the stronger interfacial interactions between the GTR and PU matrix, indicating that it has prominent wear resistance [38].

The representative stress–strain curves of PU/GTR elastomers are shown in Figure 1d, and the detailed results are shown in Table 3. Compared with the pure PUE, it can be observed that the PU/GTR elastomers exhibit an obvious strain-hardening phenomenon, possibly benefiting from the reinforcement effect of the network structure above, which was also reported in previous research [31]. Additionally, the tensile strengths of the PU/GTR elastomers are slightly increased to 36.1 MPa (PU-4) and then decrease sharply as the PCL-1K content increases, while their corresponding elongation at break shows almost no change (Figure 1d). When the PCL-1K content exceeds 59.5%, there may be an obvious reduction in the order degree in hard domains and the average molecular weight between GTR particles, which weakens the reinforcement effect derived from the hard domains [39] and the aforementioned network, explaining the lower tensile strength and elongation at break for PU-5 and PU-6.

In summary, the optimal PU/GTR thermoplastic elastomer (PU-4) not only possesses excellent wet-skid resistance and extremely low dynamic heat build-up capacities, but it also has outstanding mechanical properties and wear resistance, indicating a potential application in the fields of fuel economy and carbon dioxide reduction.

### 3.2. XPS Analysis of PU/GTR Thermoplastic Elastomers

**Figure 2:**
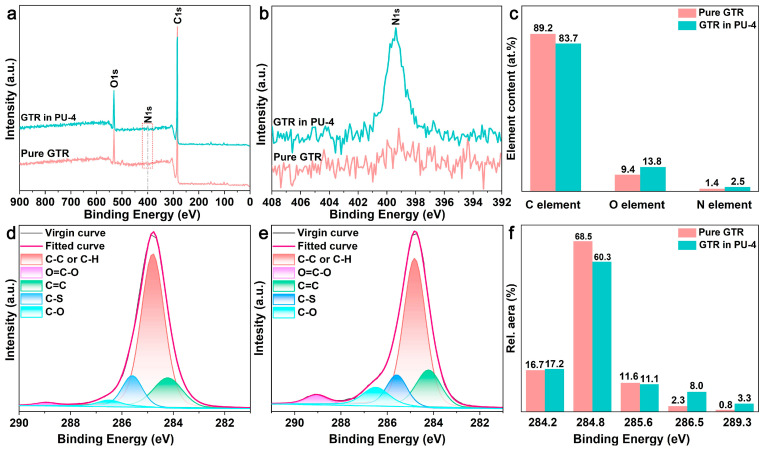
XPS survey spectrum of GTR (**a**), N1s XPS spectra of GTR (**b**), atomic ratio of different elements of GTR (**c**), C1s XPS spectra of pure GTR (**d**), C1s XPS spectra of GTR in PU-4 (**e**), the relative area obtained from the deconvoluted C1s XPS spectra of GTR (**f**).

As is well known, the uniform dispersion of fillers in the polymer matrix is determined by several factors [40,41], such as the particle size of fillers, the interfacial adhesion between fillers and polymer matrix, experimental factors, etc. Among them, the formation of the network structure described above is closely related to the strong interfacial bonding between GTR particles and the polyurethane matrix. Due to the dissolvability of PU/GTR thermoplastic elastomers (Figures S2 and S3), the treated GTR particles (GTR in PU-4), acquired from dissolving PU-4 with DMF, are employed to investigate the interfacial adhesion using XPS analysis, as shown in Figure 2. Compared with pure GTR, there is an obvious N1s signal in the survey spectrum for the GTR in PU-4 (Figure 2a,b). It can be clearly seen that the atomic contents of the N and O elements of GTR in PU-4 are, respectively, 2.5% and 13.8% higher than those in pure GRT (1.4% and 9.4%), as shown in Figure 2c. The significant increase in the above elements in the treated GTR particles may be attributed to the carbamate groups (HNCOO) on the GTR surface, which formed during the in-situ synthesis of PU/GTR elastomers.

Furthermore, the fitting results in the C1s spectra could further confirm the existence of HNCOO, which is formed through the additional reaction of hydroxyl on the GTR surface and MDI, as shown in Figure 2d,e. Compared with pure GTR (Figure 2d), it can be clearly observed that there are five characteristic peaks located at 284.2, 284.8, 285.6, 286.5 and 289.3 eV in the GTR in PU-4 (Figure 2e), attributed to the C=C, C–H/C–C, C–S, C–O and O-C=O bonds [42,43], respectively. Figure 2f displays the relative contents of each fitting peak in the C1s spectra. Obviously, the contents of C–O and O–C=O in the GTR in PU-4 are 8.0% and 3.3%, respectively, which are increased by 247.5% and 312.5% compared to those of pure GTR. Combined with the phenomenon (Figure S4) of the near-disappearance of the peak at 3300~3600 cm^−1^ (absorption peak of OH) in the FTIR spectra of pure GTR and the stronger absorption intensity of peaks at 2800~3000 cm^−1^ (vibration absorption peaks of CH_2_) and 1737 cm^−1^ (vibration absorption peak of C=O), O-C=O bonds belonging to the HNCOO group can be fully confirmed to exist on the GTR’s surface. That is to say, there is a strong chemical bond between the GTR and polyurethane matrix based on the XPS and FTIR results, indirectly evidencing the hypothesis of the network structure.

### 3.3. FTIR Analysis of PU/GTR Thermoplastic Elastomers

**Figure 3:**
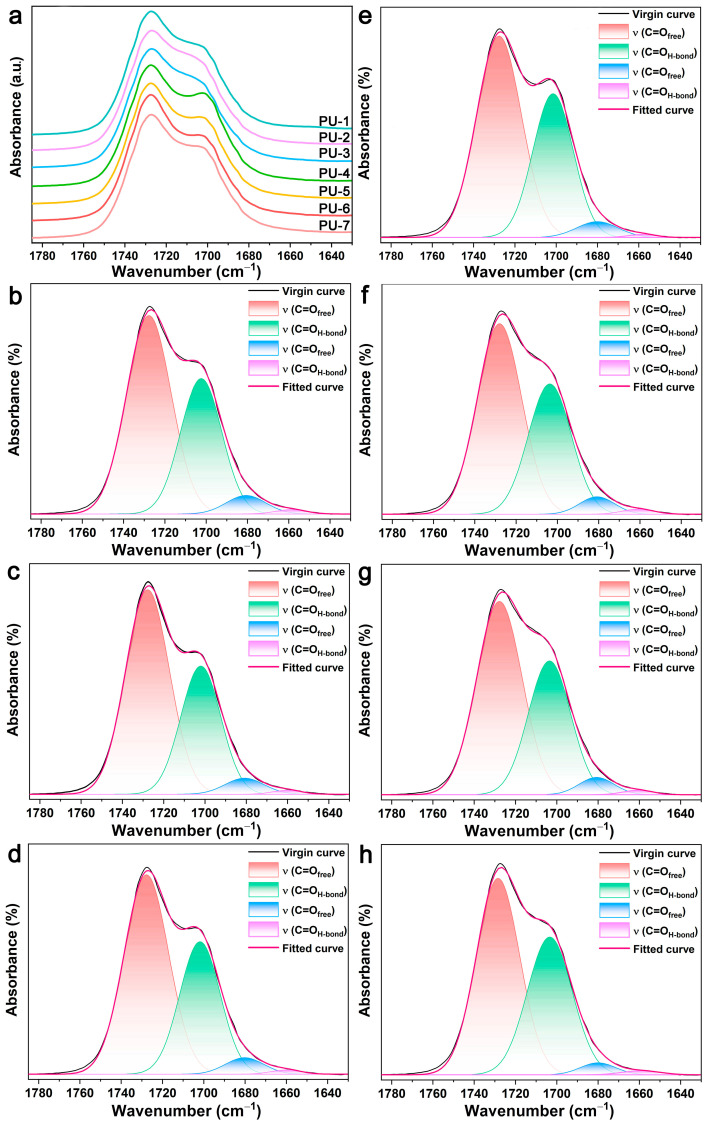
FTIR spectra of PU/GTR thermoplastic elastomers (**a**), Gaussian fitting curves of PU-1 (**b**), Gaussian fitting curves of PU-2 (**c**), Gaussian fitting curves of PU-3 (**d**), Gaussian fitting curves of PU-4 (**e**), Gaussian fitting curves of PU-5 (**f**), Gaussian fitting curves of PU-6 (**g**), Gaussian fitting curves of PU-7 (**h**).

FITR characterization (Figure S5) is also here employed to investigate the degree of microphase separation of PU/GTR elastomers, so as to indirectly explore the effects of PCL-1K content on the heat build-up and wet-skid resistance. Importantly, the Gaussian peak fitting curves of the spectra within the wavenumber range of 1630~1790 cm^−1^ are shown in Figure 3. The partial fitting results and the degree of hydrogen bonding association (HBA) are displayed in Table 4. In comparison, the degree of HBA of PU-4 is 39.0%, which is lower than the 42.1% of PU-7, implying that the incorporation of GTR reduces the degree of microphase separation in the polyurethane matrix [44]. Theoretically, the polyurethane with the higher degree of microphase separation always presents lower dynamic heat build-up. However, PU-4 yields lower dynamic heat generation compared with PU-7 (Figure 1a), which is possibly explained by the strong restrictive effect on polyurethane chains caused by the network structure formed via GTR particles, significantly reducing the frictional heat of polyurethane chains under alternating loads. In addition, the degree of HBA of the PU/GTR elastomers gradually increases as the PCL-1K content increases (Table 4), which does not necessarily indicate an increase in the degree of microphase separation of the polyurethane. The possible reason is attributed to the increase in the amount of hydrogen-bonded C=O groups originating from the ester groups in PCL-1K polyol with almost the same polyol contents (Table 1). On the contrary, the degree of microphase separation of PU/GTR elastomers decreases as the PCL-1K content increases, and the specific reasons will be discussed in detail below.

### 3.4. Microphase Structure Analysis of PU/GTR Thermoplastic Elastomers

**Figure 4:**
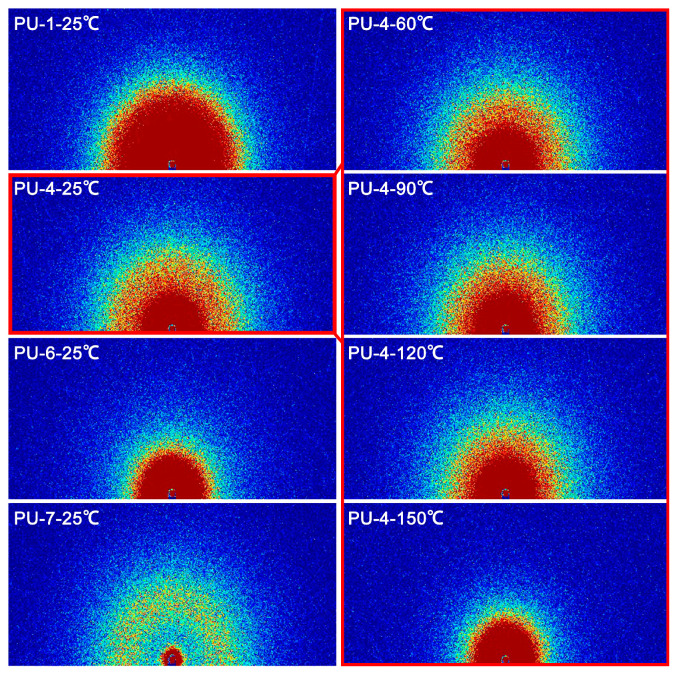
2D-SAXS patterns of PU-1, PU-4, PU-6 and PU-7 at different temperatures.

In order to further clarify the mechanisms imparting high wet-skid resistance and low heat build-up, the microphase structures of PU/GTR thermoplastic elastomers are also here investigated via SAXS, DSC and rheological characterization. Figure 4 exhibits the two-dimensional (2D) SAXS patterns of PU/GTR elastomers at different temperatures, and the equivalent radial line profiles obtained from the 2D SAXS patterns are shown in Figure S6. Compared with PU-4, PU-7 presents a more obvious semicircular scattering halo (Figure 4) and more distinct scattering peak (Figure S6), indicating that PU-7 has a higher degree of microphase separation and a randomly oriented microphase [44], which strongly supports the aforementioned conclusion that the addition of GTR weakens the microphase separation of polyurethane. PU-1 and PU-4 also display similar scattering halos, while PU-6, with lower microphase separation, has almost no such halo (Figure 4). In PU-4, the scattering halo disappears when the test temperature surpasses 120 °C, which indicates that the ordered region in the PU/GTR elastomer is related to hard segments because of the melting temperature of the soft domains being below 60 °C [45].

**Figure 5:**
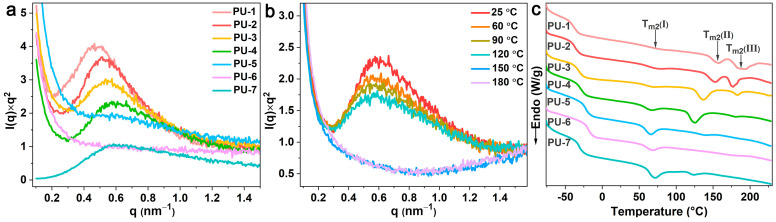
Lorentz-corrected SAXS scattering curves at 25 °C (**a**), Lorentz-corrected SAXS scattering curves during heating process (**b**), and DSC curves (**c**) of PU/GTR thermoplastic elastomers.

**Figure 6:**
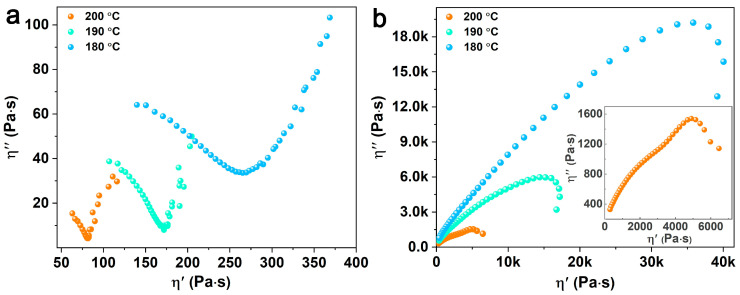
Cole–Cole plots for PU-7 (**a**) and PU-4 (**b**).

It is difficult to evaluate the interdomain distance and the degree of ordered arrangement of hard segments in PU/GTR elastomers using only the 2D-SAXS patterns and the undistinguished 1D-SAXS scattering curves. Therefore, we have further performed Lorentz correction on the I-q curves (Figure S6) to explore the microphase separation structure of PU/GTR elastomers in detail, and the relevant results are shown in Figure 5. Obviously, there is only one kind of scattering peak (Figure 6a), corresponding to the ordered domains of hard segments present in both pure PUE and PU/GTR elastomers [46], which further demonstrates that the soft segments in elastomers are transformed into an amorphous state.

Moreover, the scattering vector ($q_{max}$) at the scattering peak displays a change from 0.50 to 0.61 nm^−1^ (corresponding to PU/GTR elastomers from PU-1 to PU-6), as it gradually increases with the increasing PCL-1K content, which implies that the distance d ($d=\frac{2\pi }{q}$) between the hard domains is reduced from about 12.57 to 10.30 nm. Particularly, the scattering peak intensity decreases continuously as the PCL-1K content increases, indicating the gradual disordering of hard domains. That is to say, the increase in PCL-1K content reduces the degree of microphase separation in the polyurethane matrix, resulting in the gradually more disordered arrangement of hard segments distributed in the amorphous soft domains.

Accordingly, the special structure and distribution of hard domains have a strong constraining effect on the thermal activity of soft segments in polyurethane. Moreover, the higher the amount of PCL-1K, the stronger the constraining effect will be, which could enhance the T_g_ (Figure 1a) and tanδ at 0 °C of PU/GTR elastomers, especially for the PU-4 sample. In Figure 5b, we see a distinct reduction in the scattering peak intensity in PU-4 after increasing the test temperature, and the peak finally disappears after 120 °C, firmly proving that the ordered region is assigned to the hard domains instead of soft domains.

The ordered structure in hard domains was indirectly investigated through DSC characterization, and the results are shown in Figure 5c and Table 5. It is obvious that the T_g_ values of PU/GTR elastomers increase from −39.4 to −18.5 °C, which is consistent with the DMA results. Owing to the weak microphase separation, the T_g_ of PU-4 is increased to 1.3 °C, higher than that of its counterpart PU-7 (−31.6 °C) (Table 5). Additionally, there are three endothermic thermal behaviors around 50~90 °C (T_m2_(I)), 110~170 °C (T_m2_(II)) and 170~210 °C (T_m2_(III)), possibly corresponding to the melting of the hard segments in the short-range ordered structure [47], the long-range ordered structure and the microcrystalline structure [48,49], respectively. The phenomena of the gradual disappearance of the melting peak in microcrystalline hard segments and the decrease in T_m2_(II) demonstrate that the ordering of hard segments is seriously hindered after adding PCL-1K polyol, and only the short-range ordered structure is formed in hard segments when the PCL-1K content exceeds 80%, which effectively confirms our hypothesis, as described in the DMA analysis.

Given that PU-7 and PU-4 exhibit similar results in their SAXS and DSC characterizations, rheological measurements are employed to further investigate the microphase separation via Cole–Cole plots at different temperatures (180, 190 and 200 °C), as displayed in Figure 6. As seen in Figure 6a, the Cole–Cole plots of PU-7 are composed of a vague semicircle (low viscosity) and a rigid tail (high viscosity). The behavior is also more obvious at low temperatures, which demonstrates that there is a micro-phase separation structure in pure PUE. In PU-4, it is found that the Cole–Cole curves are markedly different from those of the pure PUE, exhibiting a single relaxation arc, especially at 180 °C (Figure 6b). Other PU/GTR elastomers also display similar plots (Figure S7a). This indicates that GTR particles have a prominent inhibitory effect on the mobility of molecular chains in hard segments, acting via strong interfacial adhesion (obtained from XPS analysis), thus weakening the degree of micro-phase separation in PU/GTR elastomers, which is consistent with the FTIR results. In addition, the terminal slope of ${lgG}^{\prime }$−lgω plots at low frequency (Figure S7b) for PU-4 is 1.20, which is higher than the value of 0.74 seen for PU-7. Obviously, the slope of PU-4 is much closer to 2, regarded as the typical terminal slope of linear polymers [50,51]. The values of PU-1 (1.16), PU-2 (1.01), PU-3 (1.12), PU-5 (1.22), and PU-6 (1.33) are also higher than that of pure PUE, indicating reduced microphase separation in the PU/GTR elastomers [52,53]. This indirectly proves the uniform distribution of the GTR particles in the polyurethane matrix. If the distribution of GTR particles in the PU matrix is not uniform, the Cole–Cole plots of PU/GTR elastomers will not show a relatively singular relaxation arc. As a consequence, the existence of a network structure formed by GTR particles in the PU/GTR elastomers is evidenced via the analysis of chemical bonds between GTR and the polyurethane matrix, as well as the semicircular relaxation arc of the Cole–Cole plots.

### 3.5. Stress Relaxation Analysis of PU/GTR Thermoplastic Elastomers

Considering that the PU/GTR thermoplastic elastomers exhibit a lower heat build-up as the temperature exceeds 60 °C (Figure 2b), stress relaxation experiments at 80 °C are used to further investigate the viscoelastic behavior of elastomers, which is conducive to illustrating the mechanism of the low dynamic heat build-up. The generalized Maxwell (GM) model is used to simulate the stress relaxation behavior of PU/GTR elastomers based on Equation (1). The fitting results of the experimental data for PU-7 and PU-4 specimens are shown in Figure S8. It is obvious that the GM model composed of three Maxwell units (Figure S9) can effectively simulate the stress relaxation behavior of both PU-7 and PU-4, with the Adj. R^2^ being 0.99994 and 0.99993, respectively.

(1) $$\frac{\sigma_{t}}{\sigma_{0}}=\frac{{\varepsilon E}_{\infty }}{\sigma_{0}}+\frac{\varepsilon }{\sigma_{0}}\sum_{i=1}^{n}E_{i}e^{-\frac{t}{\tau_{i}}}$$

where $\sigma_{t}$ is the tensile stress during the stress relaxation experiment, $\sigma_{0}$ is the tensile stress at the beginning of the stress relaxation experiment, $E_{\infty }$ is the modulus of the parallel spring, *t* is the deformation time, ε is 7.0%, and $\tau_{i}$ and $E_{i}$ correspond to the relaxation time and the relaxation modulus of the *i*-th Maxwell unit, respectively.

**Figure 7:**
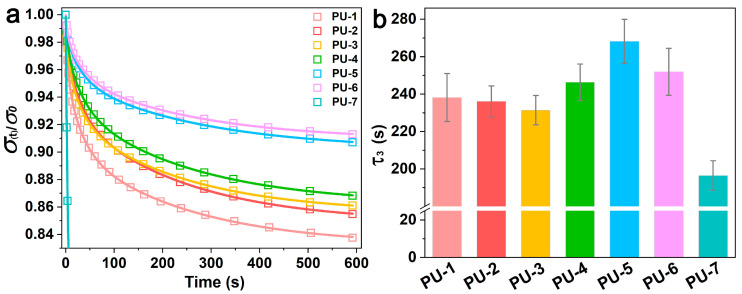
Modeling of experimental stress relaxation curves (**a**) and relaxation times ($\tau_{3}$) (**b**) for PU/GTR thermoplastic elastomers.

Consequently, the GM model of three Maxwell units is employed to simulate the stress relaxation behaviors of the PU/GTR elastomers via Equation (2), and the results including equilibrium modulus, relaxation time and Adj. R^2^ are shown in Figure 7 and Table S1.

(2) $$\frac{\sigma_{t}}{\sigma_{0}}=\frac{{\varepsilon E}_{\infty }}{\sigma_{0}}+\frac{{\varepsilon E}_{1}}{\sigma_{1}}e^{-\frac{t}{\tau_{1}}}+\frac{{\varepsilon E}_{2}}{\sigma_{2}}e^{-\frac{t}{\tau_{2}}}+\frac{{\varepsilon E}_{3}}{\sigma_{3}}e^{-\frac{t}{\tau_{3}}}$$

It can be seen that the stress relaxes to a finite value over the observation time period for all samples, i.e., the equilibrium modulus ($E_{\infty }$) of both pure PUE and PU/GTR elastomers reduces to a certain value (>0), which may be due to the existence of cross-linked networks. It should be mentioned that the $E_{\infty }$ values of PU/GTR elastomers are significantly larger than 0.20 MPa for pure PUE, which could be exclusively explained by the simple physical cross-linked networks with ordered hard domains in PU-7. However, PU/GTR elastomers have two types of mutually restrictive cross-linked networks, namely, a physical network composed of hard domains and a network derived from GTR particles, leading to the higher $E_{\infty }$ of more than 5.06 MPa (Table S1). Furthermore, Maxwell unit 1 ($E_{1}$, $\tau_{1}$), Maxwell unit 2 ($E_{2}$, $\tau_{2}$) and Maxwell unit 3 ($E_{3}$, $\tau_{3}$) are associated with the conformational changes and the uncoiling of molecular chains in amorphous PCL domains [54], PCL molecular chains between the ordered hard domains and molecular chains in hard segments distributed in amorphous PCL domains, respectively. Notably, the $\tau_{3}$ of PU/GTR elastomers is significantly higher than the 196.46 s of pure PUE, especially for the PU-4 (246.3 s), as shown in Figure 7b. Compared with the easily conformational changes and uncoiling of PCL molecular chains, the molecular chains in hard segments exhibit lower mobility in PU-4, thus displaying a lower ${\;\tau }_{3}$. The other relaxation times and equilibrium moduli (Table S1) are discussed in the Supplementary Material. Moreover, the GTR particles in PU/GTR elastomers interact with the molecular chains of hard segments distributed in amorphous PCL domains through carbamate bonds, further reducing the mobility of the molecular chains in PU/GTR elastomers. Accordingly, it can be reasonably inferred that the above-mentioned hard segments and GTR particles in networks (obtained from rheological property analysis) can collaboratively act as “anchor points” under alternate loads for samples, thus reducing the frictional heat generated from molecular chains sliding, which endows the PU/GTR elastomers with lower heat build-up.

## 4. Conclusions

In this study, eco-friendly high-performance PU/GTR thermoplastic elastomers are successfully prepared by the in-situ synthesis method. When the amount of PCL-1K is 59.5%, the PU/GTR elastomer shows a prominent comprehensive performance with a tanδ at 0 °C, tanδ at 60 °C and ∆rel of 0.92, 0.03 and 38.5 mm^3^, respectively, which demonstrates that the elastomer simultaneously possesses high wet-skid resistance, low rolling resistance and high wear resistance. The results show that the increase in PCL-1K causes the PU/GTR elastomer to exhibit a gradual decrease in the interdomain distance between the hard domains and the degree of orderliness in hard domains, thus leading to an increase in the T_g_ of PU/GTR elastomers. Moreover, GTR is uniformly distributed in the polyurethane matrix in the form of a network through the strong interfacial bonds formed during in-situ polymerization. The network has a strong restrictive effect on polyurethane chains, thus reducing the frictional heat generated from molecular chains’ sliding, which endows the PU/GTR elastomers with a lower heat build-up capacity. Meanwhile, a similar inhibitory effect is also conducive to improving the tanδ at 0 °C of the PU/GTR elastomer. Ultimately, the goal of the controllable adjustment of tanδ at 0 °C has been realized, resulting in the successful preparation of the PU/GTR elastomer with high wet-skid resistance. Besides this, the strong interfacial strength enables the PU/GTR elastomer to exhibit excellent wear resistance. In summary, this study not only provides novel ideas for the preparation of polyurethane tires, but it also provides a potential approach to the utilization of waste tire rubber.

## Figures and Tables

**Table 1:** The formulation used to synthesize PU/GTR thermoplastic elastomers.

Samples	^1^ PCL-1K Content (%)	PCL-1K (g)	PCL-2K (g)	GTR (g)	MDI (g)	BDO (g)
PU-1	0	0	190	10	75	15.9
PU-2	20.5	39	151	10	75	14.2
PU-3	39.5	75	115	10	75	12.6
PU-4	59.5	113	77	10	75	10.9
PU-5	80.0	152	38	10	75	9.1
PU-6	100	190	0	10	75	7.4
PU-7	59.5	119	81	0	75	12.6

^1^ PCL-1K content refers to the proportion of PCL-1K in total polyols.

**Table 2:** T_g_, tanδ at 0 °C and tanδ at 60 °C of PU/GTR thermoplastic elastomers.

Sample	T_g_ (°C)	tanδ at 0 °C	100 × tanδ at 60 °C
PU-1	−12.1	0.45	2.59
PU-2	−7.9	0.63	2.63
PU-3	−2.2	0.80	2.65
PU-4	1.9	0.92	3.04
PU-5	12.0	0.49	2.47
PU-6	11.7	0.42	1.85
PU-7	2.0	0.80	11.77

**Table 3:** Mechanical properties of PU/GTR thermoplastic elastomers.

Sample	Tensile Strength (MPa)	Elongation at Break (%)	Tensile Modulus (MPa)	Hardness (Shore A)
PU-1	30.0 ± 0.3	447.7 ± 16.1	19.3 ± 0.4	83
PU-2	30.7 ± 0.3	431.6 ± 10.6	19.2 ± 0.8	80
PU-3	35.3± 0.4	434.2 ± 17.0	17.4 ± 0.8	78
PU-4	36.1 ± 0.6	450.0 ± 17.9	13.8 ± 0.6	78
PU-5	25.0 ± 0.3	430.9 ± 18.3	10.0 ± 1.0	70
PU-6	21.0 ± 0.5	384.1 ± 9.9	8.4 ± 0.8	62
PU-7	9.0 ± 0.5	492.2 ± 16.6	8.5 ± 0.2	70

**Table 4:** The partial fitting results and the degree of HBA of PU/GTR thermoplastic elastomers.

Sample	Adj. R^2^	Area Integral (Wavenumber, cm^−1^)		The Degree of HBA (%)
PU-1	0.9995	6.77 (1702.4)	10.74 (1727.7)	38.7
PU-2	0.9994	6.21 (1702.3)	10.87 (1727.8)	36.4
PU-3	0.9995	6.35 (1701.8)	10.53 (1727.8)	37.6
PU-4	0.9995	6.86 (1701.5)	10.75 (1727.7)	39.0
PU-5	0.9995	6.66 (1703.7)	10.07 (1728.0)	39.8
PU-6	0.9995	6.72 (1703.5)	10.40 (1727.8)	39.3
PU-7	0.9994	7.33 (1703.4)	10.10 (1728.5)	42.1

**Table 5:** DSC data of polyurethane/GTR thermoplastic elastomer.

Samples	T_g_ (°C)	T_m_(I) (°C)	ΔH_m_(I) (J/g)	T_m_(II) (°C)	ΔH_m_(II) (J/g)	T_m_(III) (°C)	ΔH_m_(III) (J/g)
PU-1	−39.4	78.9	0.6	156.8	2.3	191.2	5.2
PU-2	−37.3	74.4	1.6	152.4	2.9	177.0	3.2
PU-3	−32.1	68.7	1.1	136.6	5.0	182.9	1.1
PU-4	−30.3	66.2	2.1	125.3	5.1	180.1	0.5
PU-5	−22.5	65.7	3.4	138.3	0.7	-	-
PU-6	−18.5	67.8	2.4	-	-	-	-
PU-7	−31.6	71.2	4.7	123.8	0.8	-	-

## Data Availability

The data presented in this study are available on request.

## Supplementary Materials

The following supporting information can be downloaded at: https://www.mdpi.com/article/10.3390/polym16172448/s1, Figure S1: Particle size distribution of GTR; Figure S2: Dissolved PU-4 (a_1_~a_6_) and PU-7 (b_1_~b_2_) in DFM solvent a_1_~a_6_—12 h, 24 h, 36 h, 48 h, 60 h, 72 h; b_1_~b_2_—12 h, 24 h; Figure S3: MFR for PU/GTR thermoplastic elastomers; Figure S4: FTIR spectra of pure GTR and GTR components in PU-4; Figure S5: FTIR spectra of PU/GTR thermoplastic elastomers; Figure S6: 1D-SAXS scattering curves of PU/GTR thermoplastic elastomers (a) and 1D-SAXS scattering curves of PU-4 at different temperature (b); Figure S7: Cole–Cole plots (a) and G′-ω curves (b) at 180 °C for PU/GTR elastomers; Figure S8: Modeling of experimental stress relaxation curves for PU-7 (a) and PU-4 (b); Figure S9: The generalized Maxwell (GM) model; Table S1: The fitting parameter values of the GM model using Equation (2) for the experimental stress relaxation data for PU/GTR elastomers.
