# Supplementary material for: Novel Design of Eco-Friendly High-Performance Thermoplastic Elastomer Based on Polyurethane and Ground Tire Rubber toward Upcycling of Waste Tires
Source: Polymers (Basel). 2024 Aug 29;16(17):2448. doi: 10.3390/polym16172448 (PMC11398027; doi:10.3390/polym16172448)
Supplement: Supplementary file 1 [file polymers-16-02448-s001.zip › Supplementary material-Figure S2.pdf]

Firstly, the PU/GTR elastomer was immersed in DMF solvent for 72 h (as shown in Figure S2). And then the GTR sample was obtained by centrifugation treatment (7000 rpm, 10 min) after repeated cleaning of DMF.

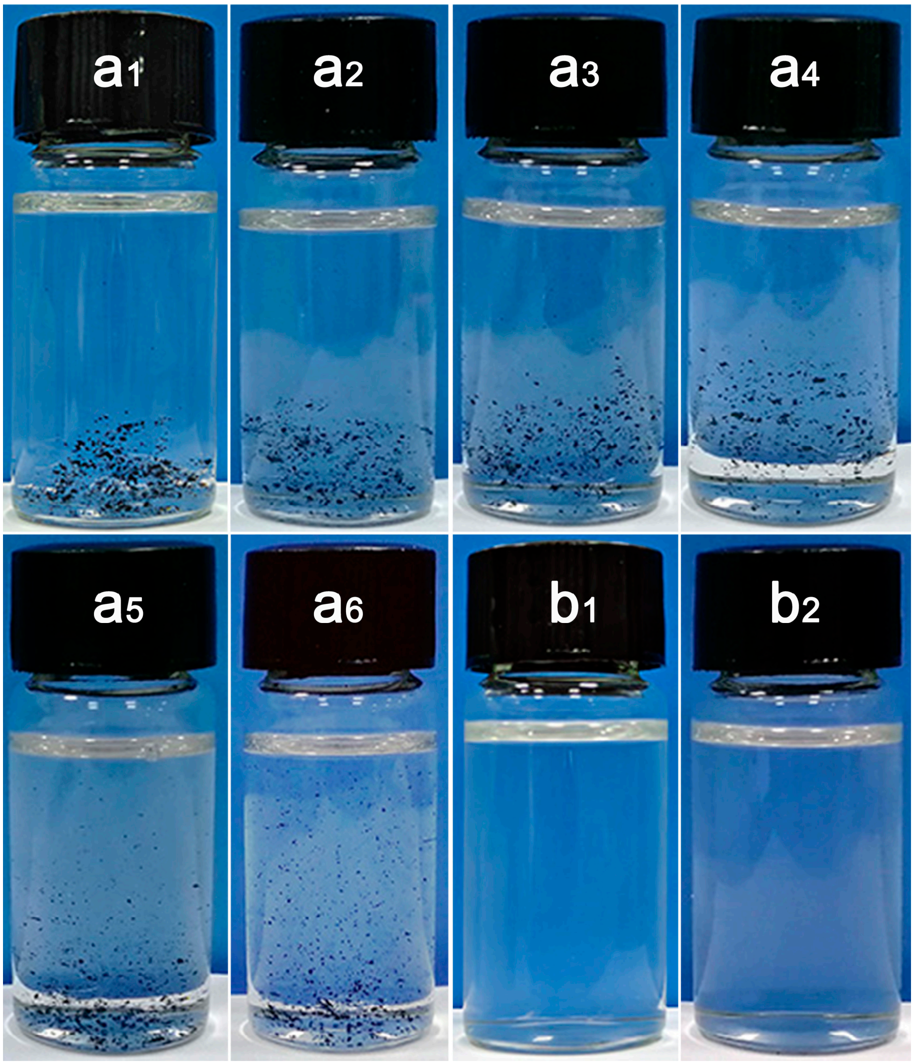

Figure S2. Dissolved PU-4 (a_1~a_6) and PU-7 (b_1~b_2) in DFM solvent: a_1~a_6: 12 h, 24 h, 36 h, 48 h, 60 h, 72 h; b_1~b_2: 12 h, 24 h

Due to absence of the “crosslinked network”, the pure polyurethane (PU-7) was completely dissolved in DMF solvent within 24 hours (Figure S2 b_1~b_2) under the same conditions. However, it needed 72 hours for the PU/GTR elastomer (PU-4) to completely dissolve in DMF solvent, which indirectly proved that there was “crosslinked network” indeed existed in polyurethane matrix.
