# Supplementary material for: Novel Design of Eco-Friendly High-Performance Thermoplastic Elastomer Based on Polyurethane and Ground Tire Rubber toward Upcycling of Waste Tires
Source: Polymers (Basel). 2024 Aug 29;16(17):2448. doi: 10.3390/polym16172448 (PMC11398027; doi:10.3390/polym16172448)
Supplement: Supplementary file 1 [file polymers-16-02448-s001.zip › Supplementary material-Figure S3.pdf]

The melt flow rate (MFR) of PU/GTR thermoplastic elastomers was tested using the melt flow rate meter (GOTECH Testing Machines Co., Ltd., GT-7100-MI, China). The test temperature is 200 ℃ with the load being 2.5 kg and the MFR of PU/GTR elastomers is shown in FigureS3. Specially, the MFR of pure PU is too high to record.

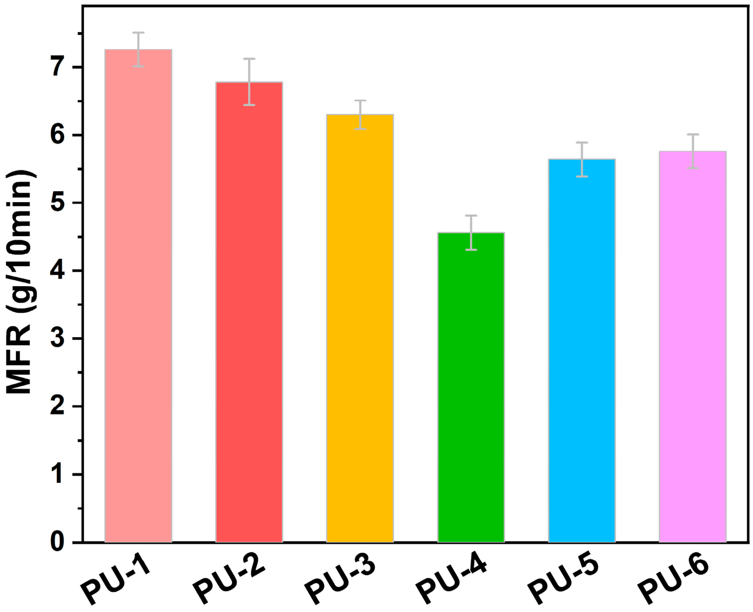

Figure S3. MFR of for PU/GTR thermoplastic elastomers.
