# Supplementary material for: Novel Design of Eco-Friendly High-Performance Thermoplastic Elastomer Based on Polyurethane and Ground Tire Rubber toward Upcycling of Waste Tires
Source: Polymers (Basel). 2024 Aug 29;16(17):2448. doi: 10.3390/polym16172448 (PMC11398027; doi:10.3390/polym16172448)
Supplement: Supplementary file 1 [file polymers-16-02448-s001.zip › Supplementary material-Figure S4.pdf]

The GTR pellets for testing were prepared via the mechanical pressing method with the mass ratio of potassium bromide to GTR is 200:1.

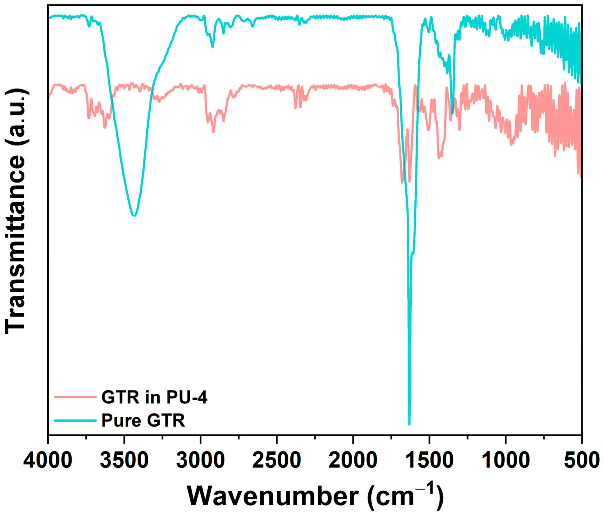

Figure S4. FTIR spectra of pure GTR and GTR components in PU-4
