# Supplementary material for: Novel Design of Eco-Friendly High-Performance Thermoplastic Elastomer Based on Polyurethane and Ground Tire Rubber toward Upcycling of Waste Tires
Source: Polymers (Basel). 2024 Aug 29;16(17):2448. doi: 10.3390/polym16172448 (PMC11398027; doi:10.3390/polym16172448)
Supplement: Supplementary file 1 [file polymers-16-02448-s001.zip › Supplementary material-Figure S6.pdf]

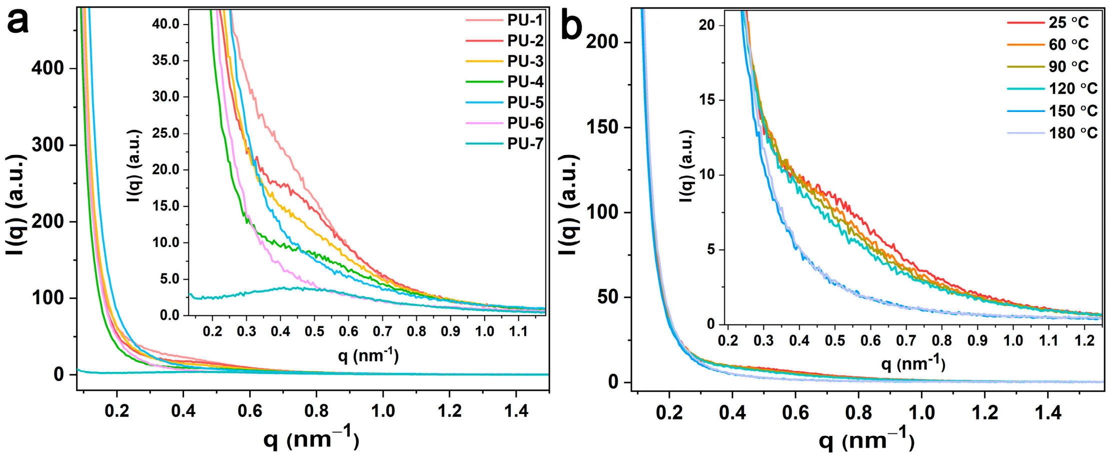

Figure S6. 1D-SAXS scattering curves of PU/GTR thermoplastic elastomers (a) and 1D-SAXS scattering curves of PU-4 at different temperature (b)
