# Supplementary material for: Novel Design of Eco-Friendly High-Performance Thermoplastic Elastomer Based on Polyurethane and Ground Tire Rubber toward Upcycling of Waste Tires
Source: Polymers (Basel). 2024 Aug 29;16(17):2448. doi: 10.3390/polym16172448 (PMC11398027; doi:10.3390/polym16172448)
Supplement: Supplementary file 1 [file polymers-16-02448-s001.zip › Supplementary material-Table S1.pdf]

Table S1. The fitting parameter values of GM model using Equation (2) for the experimental stress relaxation data for PU/GTR elastomers.

Samples	E_∞ (MPa)	E_1 (MPa)	τ_1 (s)	E_2 (MPa)	τ_2 (s)	E_3 (MPa)	τ_3 (s)	Adj. R^2
PU-1	8.11	0.39	4.05	0.53	29.31	0.73	238.19	0.99990
PU-2	10.18	0.26	4.04	0.58	28.23	0.97	236.10	0.99994
PU-3	9.17	0.30	4.30	0.48	27.20	0.76	231.46	0.99994
PU-4	6.28	0.16	4.45	0.31	31.76	0.54	246.30	0.99994
PU-5	5.02	0.09	4.40	0.18	31.84	0.29	268.20	0.99993
PU-6	5.06	0.08	4.58	0.16	34.26	0.27	251.95	0.99992
PU-7	0.20	0.11	4.20	0.17	27.44	0.23	196.46	0.99993

As well known, an increase of temperature will accelerate the stress relaxation of polymer chains. Thus, the τ_1 is less than 5 seconds because of the melting temperature of PCL being much lower than test temperature (80 ℃). And τ1 increases gradually with the PCL-1K content increasing, which may be related to the weakened restrictive effect of lower microphase separation on the soft segment molecular chains, meanwhile, the E_1 constantly decreases. Additionally, the decrease of the interdomains (obtained in SAXS analysis) makes the uncoiling of PCL molecular chains between hard domains much easier, resulting in the reduce of the τ_2. However, the binding effect on the PCL molecular chains that caused by the reduction degree of ordering in the hard domains begin to weaken as the PCL-1K content higher than 59.5%, leading to an extension of τ_2. Due to the reinforcement of the hard segments, the E_2 is higher than that of Maxwell unit 1, but also shows a gradually decreasing trend.
