# Supplementary figures and images for: Novel Design of Eco-Friendly High-Performance Thermoplastic Elastomer Based on Polyurethane and Ground Tire Rubber toward Upcycling of Waste Tires
Source: Polymers (Basel). 2024 Aug 29;16(17):2448. doi: 10.3390/polym16172448 (PMC11398027; doi:10.3390/polym16172448)

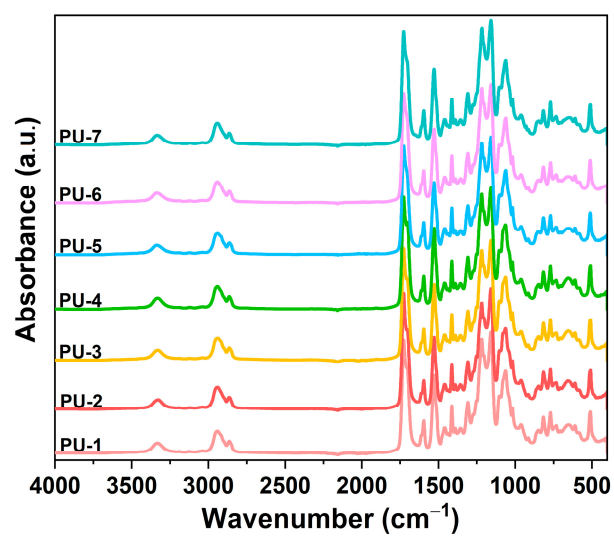

Figure S5. FTIR spectra of PU/GTR thermoplastic elastomers

Supplement: Supplementary file 1 [file polymers-16-02448-s001.zip › Supplementary material-Figure S5.pdf]

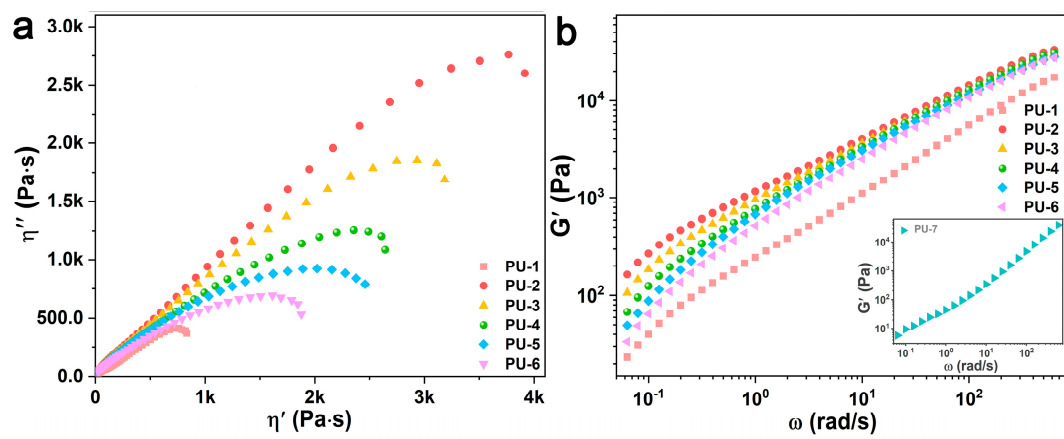

Figure S7. Cole-Cole plots (a) and  $G'$ - $\omega$  curves (b) at 180 °C for PU/GTR elastomers.

Supplement: Supplementary file 1 [file polymers-16-02448-s001.zip › Supplementary material-Figure S7.pdf]

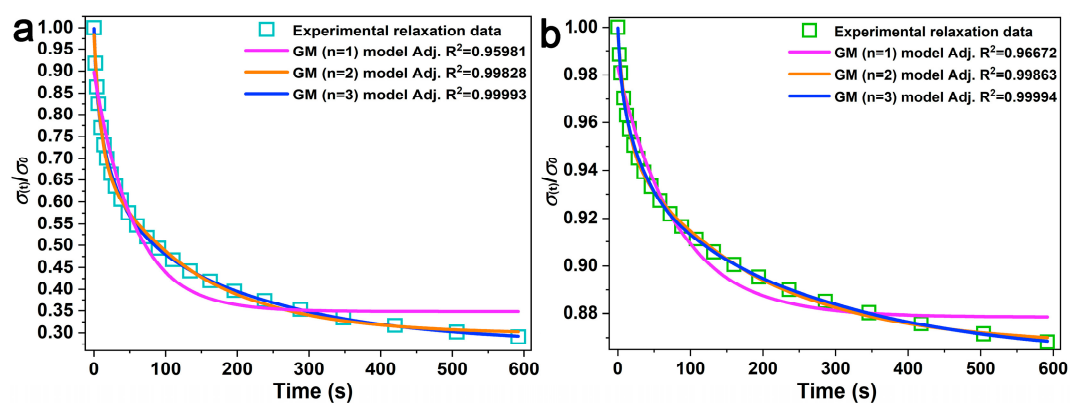

**Figure S8.** Modeling of experimental stress relaxation curves for PU-7 (a) and PU-4 (b).

Supplement: Supplementary file 1 [file polymers-16-02448-s001.zip › Supplementary material-Figure S8.pdf]

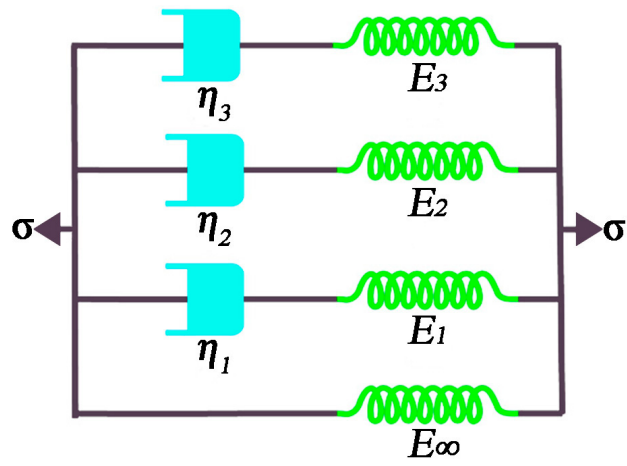

**Figure S9.** The generalized Maxwell (GM) model.

Supplement: Supplementary file 1 [file polymers-16-02448-s001.zip › Supplementary material-Figure S9.pdf]
